# Supplementary material for: Content-rich biological network constructed by mining PubMed abstracts
Source: BMC Bioinformatics. 2004 Oct 8;5:147. doi: 10.1186/1471-2105-5-147 (PMC528731; doi:10.1186/1471-2105-5-147)
Supplement: Additional File 2 — The original results of the above study (non-essential files are deleted to keep the file size under the limit set by BMC bioinformatics). [file 1471-2105-5-147-S2.bz2 › chilibotAdditionalFile2/dip05/510ID11117529E10805/html/ESR1_ZNF151.html]

 


 **ESR1** and **ZNF151** 
  
Found 2 abstracts in PubMed, retrieved 2.  
 

 What does Google say? 
 PDF only 
| .edu only 

---

**Interactive relationship** (e.g. stimulation, inhibition, etc)

**Stimulatory relationship**- ARIP3 enhances glucocorticoid receptor dependent transcription more efficiently than Miz1  [ **ZNF151** ]  or PIAS1, and all PIAS proteins also activate estrogen receptor  [ **ESR1** ]  and progesterone receptor dependent transcription but to a dissimilar degree.  Ref: 11117529 Mol Endocrinol, 2000
**Neutral relationship**
